# Everybody's Page

**Published:** 1889-01-26

**Authors:** 


### Everybody's Page.

The compiler of "News in a Nutshell" in the *Echo* of the 19th inst. has a tender conscience. He took seven items from this page last week, and gave us credit for one. But why one? Why not all or none?

---

There are in the United States no fewer than 563 manufactories of patent medicines, of which 108 are in the state of New York alone.

---

"It is an ill wind that blows nobody good." It is well-known that the Emperor of Germany suffers from aural disease. During the last five weeks twenty-five pamphlets on ear troubles have issued from the German press. The Emperor may not profit by them; but the printers do.

---

Professor Oscar Schmidt, of Gratz, in Styria, has suggested that sponges could be propagated by fixing pieces detached from living sponges to sandy coasts by means of wooden skewers, and leaving them to grow. The Austro-Hungarian Government has recently taken steps to protect the sponge industry on the shores of Dalmatia, reckless gathering having threatened extermination.

---

The old Greek belief was that Æsculapius, the first great physician, and Chiron, the first great surgeon, were both killed by the thunderbolts of Jove, because they saved too many lives, and even raised men from the dead. But as a recognition of their merits, he set them among the stars, translating one into the constellation of Orion, and the other into that of the Centaur.

---

A commission charged by the French Government with the duty of investigating the evils resulting from the consumption of alcohol, set down as causes of equal danger the abuse of intoxicants and the impure nature of many of them. The employment of what they term "alcools d'industrie"—in familiar phrase, "doctored spirits," in place of the pure product of fermentation is proving a serious danger to the health and prosperity of the nation.

---

A French doctor named Rengade has just published a novel, "Le Docteur Fabrice," the hero of which is a medical man, who practices his vocation on the battle-fields of the war of 1870, and notes with professional interest the symptoms of his own death malady. After depicting the heroic life and work of Fabrice, Dr. Rengade his regret that "in a profession so full of perils as medicine, where the doctor dies like the soldier, the State takes no trouble about those whom he leaves behind him."

---

When China is conquered in any matter of progress, the world is conquered. Therefore it may be taken as a proof of the universal acceptance of scientific medicine that the first professional examination in the Hong Kong College of Medicine for the Chinese took place a little time ago. The subjects of examination were botany, chemistry, physics, elementary anatomy, elementary physiology, materia medica, and clinical observations. Twelve students presented themselves for examination, of whom seven passed. Two scholarships, worth £12 a year, were awarded to the first students.

---

Satires on the fatal results of the doctor's attention are by no means new. Here is one assigned to Nicarchus, a writer who lived in the first century of our era: "Alexis, a physician, gave a prescription to five patients, bled five more, and to five others gave an ointment. And for all there has been one night, one coffin-maker, one grave, one Hades, one lamentation." There are some jokes which never seem to pall on certain people. The "fine old crusted" aspersion of the obnoxious mother-in-law is one; this of the death-dealing doctor is another.

---

There has long been a popular belief that the greatest number of deaths occur between four and six o'clock in the morning. Dr. Charles Féré has taken the trouble to tabulate the death-hours of all patients dying in two Parisian hospitals during the last ten years. He found that there were rather fewer deaths between seven and eleven o'clock in the evening than at any other time, but that there was no special preponderance at any hour.

---

Women are now permitted to work as druggists in Russia if they have obtained the necessary diploma. No proprietor of a chemist's shop, however, is permitted to have both male and female assistants on his premises. Is this because the Russian Government believes that "Two of a trade won't agree," or for the opposite reason? Certainly a flirtation that might have no serious results if it caused only a mistake in the measuring of a ribbon, may be very fatal when the work neglected or ill-done is the measuring of a drug.

---

A case that tends to show that consumption is contagious is narrated by the German doctor, Reich. A consumptive nurse in a small town was in the habit of insufflating the lungs of new born children with her own breath if there was any sign of asphyxia. Ten children whom she tended from their birth, and in whose families no predisposition existed, died of tubercular meningitis. The other nurse of the district, who used the same means to promote respiration, but who was herself healthy, had no such cases.

---

There is every probability that the cry, "What shall we do with our girls?" will soon be echoed from Germany. Recent statistics show that the number of men is decreasing relatively to that of women, and indeed there is an absolute decrease in the male population. To a nation that looks upon the army as the foundation of national prosperity this is no slight evil, but there is some consolation in the thought that their numerical predominance will probably force into larger exercise the faculties, hitherto rather cramped, of German women.

---

We have spoken more than once of the increasing prevalence of the morphia habit, especially among women. It would surely be a sufficient warning to those who have yielded to this fatal craving to learn, on the authority of Dr. Erlenmeyer, that the children of women who take morphia are practically morphia-eaters at birth. That is, unless morphia is administered to them from their earliest days they are apt to fall into a state of collapse similar to that which affects adult *habitués* if the drug be suddenly withdrawn, and the exhaustion is likely to end in death.

---

In a book entitled "A Bird's-Eye View of France in the Middle Ages," M. Challemel tells some interesting medical superstitions formerly prevalent in that country. To ward off fevers one might refrain from eating meat or eggs at Easter and on other festivals, or carry about a piece of human bone, or pluck and eat the first daisy found in the field. If fever had been contracted, the best cure was for the sufferer to rise early in the morning and go out into the fields, walking backwards all the time, pluck a handful of herbs, throw it behind him without looking at it, and then go home as fast as he could. The fever would then leave him and attack the devil instead. Running about a church with no aim in particular prevented pleurisy. Ear-ache was cured by the touch of a skeleton hand; headache by binding the temples with the rope by which someone had been hanged; toothache by touching with a dead man's tooth—all sufficiently ghastly remedies. The most anti-hygienic notion was, perhaps, that of the Bretons, who thought they would save their children from all evils by dressing them in a damp shirt.

